# The corrosion inhibition of carbon steel in 1 M HCl solution by *Oestrus ovis* larvae extract as a new bio–inhibitor

**DOI:** 10.1016/j.heliyon.2022.e12297

**Published:** 2022-12-17

**Authors:** Haneih Mobtaker, Mahboobeh Azadi, Maryam Rassouli

**Affiliations:** a Faculty of Materials and Metallurgical Engineering, Semnan University, Semnan, Iran; b Pathobiology Department, Shamirzad School of Veterinary Medicine, Semnan University, P. O. Box: 35131-19111, Semnan, Iran

**Keywords:** *Oestrus ovis* larvae extract, Bio–inhibitor, Corrosion properties, 1 M HCl solution, Electrochemical tests

## Abstract

Since, nowadays, utilizing the eco-friendly and economic corrosion inhibitors in various industries is a challenge, in this research, the corrosion behavior of carbon steel in the HCl solution by the addition of the extract of *Oestrus ovis* larvae as a novel bio-inhibitor has been evaluated. The electrochemical tests plus the gravimetric investigations were performed to study the corrosion property of steel substrates in various concentrations of bio-inhibitor (0.25–3 g/L). Different methods such as grazing incidence X-ray diffraction, field emission scanning electron microscopy (FESEM), and atomic force spectroscopy (AFM), were utilized to detect the chemical composition and morphology of corroded surfaces. Results of the Tafel polarization showed that the inhibition efficiency was about 57–86% with the highest value at the inhibitor concentration of 1 g/L. Electrochemical impedance spectroscopy results indicated that with the specified concentration of bio-inhibitor the electrochemical properties of samples changed based on the suggested electrical circuit. Results showed that the adsorption isotherm of the inhibitor was the Langmuir model with the cathodic-anodic performance. Both FESEM and AFM images demonstrated that the intensity of deterioration and the roughness of corroded surfaces reduced significantly at the optimum concentration of inhibitor (1 g/L). The inhibition mechanism was proposed based on experimental results.

## 1 Introduction

When metallic structures are exposed to acidic solutions in various industries, such as in acidic tanks, they were corroded due to the voltage difference between metal and hydrogen ions. In this situation, the metallic surface should be protected from degradation. There are various ways to decrease or stop the corrosion rates of metallic surfaces such as the usage of protective coating, adding the proper inhibitor, cathodic, and anodic protection [1, 2, 3]. The usage of the inhibitor is a conventional way especially for acid cleaning of various industrial parts. However, the traditional materials that are used as inhibitors are toxic and unfriendly materials to the environment. Therefore, various researchers tried to introduce eco-friendly and natural materials that can be utilized as effective corrosion inhibitors [4, 5]. They used green materials that contained tannin, aromatic and aliphatic cyclic structures, P, S, O, N elements with free electron pairs. These materials can be adsorbed on metallic surfaces and can be extracted from various plants and insects [6, 7]. Some of these research are summarized in the following paragraph, Cheng et al. [6] added the mixture of graphene and mussel adhesive proteins to the corrosive solution to protect the steel substrate against corrosion reactions. Bidi et al. [8] used the extract of *Hyalomma* tick as bio–inhibitor for carbon steel in 1 M HCl solution. The inhibition efficacy was about 84% when the inhibitor concentration was about 1 g/L. Deivanayagam et al. [9] utilized the extract of *Gymnema Sylvestre* leaves to decrease the corrosion rate of mild steels in 1 N HCl solution. Zhang et al. [10] introduced two new derivatives of chitosan as inhibitors for 1 M HCl solution. Their results showed that the presence of inhibitors caused a reduction in the corrosion rate of mild steels. Azzaoui et al. [11] utilized the extract of Gum Arabic to increase the resistance for mild steels in the HCl solution when the inhibitor concentration was 1 g/L. Zaher et al. [12] used the *Ammi visnaga* L. Lam seeds extract for change of corrosion behavior of mild steel in HCl solution. Results showed that then inhibition efficiency of 84% would be achieved in a concentration of 1 g/L. Bidi et al. [13] mitigated the corrosion rate of steel by adding *leech* extract in the acidic solution.

In this paper, as a new study, an extract of *Oestrus ovis* larvae (*OO*L) was used to decrease the corrosion rate of carbon steel substrates in the acidic solution, since this extract contained polyamide groups and chitin [14]. Besides, the process of extraction was not a cost- and time-consuming process compared to others. The *OO*L concentration was about 0.25–3 g/L the weight loss test and electrochemical impedance spectroscopy (EIS) and the Tafel polarization tests were performed to investigate the electrochemical behavior of steel in 1 M HCl solution. The utilized analysis for studying corroded surfaces contained energy-dispersive X-ray spectroscopy (EDS), Fourier transforms infrared spectroscopy-attenuated total reflection (FTIR-ATR), grazing incidence X-ray diffraction (GIXRD) and field emission scanning electron microscopy (FESEM), and atomic force spectroscopy (AFM). Additionally, the thermodynamic and kinetic study of corrosion reaction was done when *OO*L extract was added to the corrosive solution.

## 2 Experimental part

### 2.1 Materials

The sheet of low-carbon steel (Steel DIN 17100) is used as substrates. Details of the chemical composition of the utilized substrate are reported in Table 1. Prior to the electrochemical test, substrates were polished with various SiC paper to 2500 grit, washed with the ethanol solution, and then dried in ambient air.

**Table 1:** Details of chemical composition (wt %) of utilized carbon steel.

Fe	C	Si	Mn	S	P
98.95	0.20	0.25	0.50	0.05	0.05

**Figure 1:**
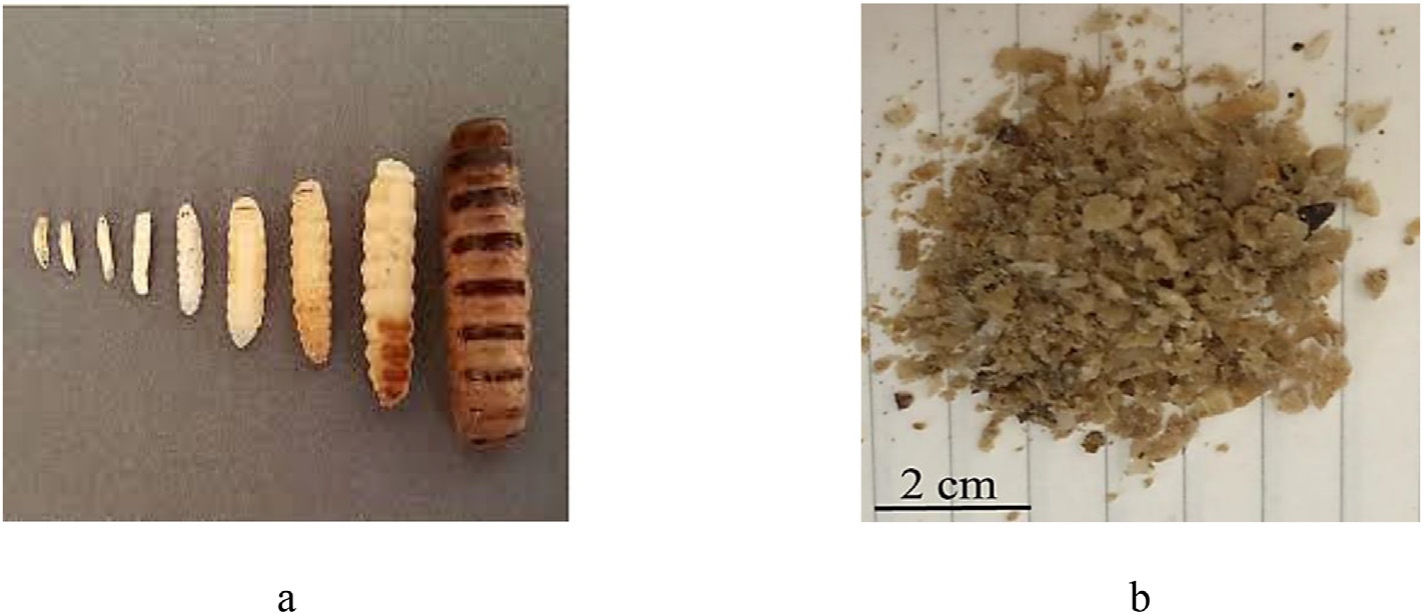
Images of (a) *Oestrus ovis* larvae (*OOL*) [15], (b) the powder extract of *OOL*.

To prepare the utilized inhibitor extract, at first *Oestrus ovis* larvae (*OO*L) was collected from the nasal sinuses of live sheep and transfer to veterinary parasitology laboratory. All procedures performed in this study involving animals were under the ethical standards of the Veterinary Faculty of Semnan University ethical committee through the received approval from the mentioned ethics committee. It was noticeable that adult female flies are ovoviviparous; they deposit newly hatched first-stage larvae that enter the nostrils of the host. The image of *OO*L evolution is shown in Figure 1(a) [15]. They were kept for 48 h in 70% ethanol for the extraction process. The parasites were then crushed with a mortar and then dried [16], as shown in Figure 1(b).

### 2.2 Methods

The concentration of inhibitor used in acidic solution was 0.25–3 g/L. More details of all samples are reported in Table 2. To detect the functional groups of the *OO*L extract, FTIR (Varian 670 IR model) method was utilized. The wavenumber was about 400–4000 cm^−1^ with a resolution of 4 cm^−1^.

**Table 2:** Details of prepared samples.

sample name	Concentration of inhibitor (g/L)
0–L	0
0.25–L	0.25
0.5–L	0.5
1–L	1
1.5–L	1.5
2–L	2
3–L	3

Prior to corrosion tests, samples were exposed to the corrosive solution in order to reach a stable condition. The open-circuit potential (OCP) for various samples were measured after 1 h. The time for this measurement was about 2800 s. For electrochemical tests, a potentiostat device (Organ-Auto-lab) plus a flat cell was used. The specimen with an area of 0.8 cm^2^, the platinum thin sheet, and the saturated calomel electrode (SCE) were the working, counter, and reference electrodes. At a temperature of 301 K, both Tafel polarization and EIS tests were used at various concentrations in 1 M HCl solution (with a pH value of 0.1). However, for the temperature range of 298–313 K, Tafel polarization tests were performed when the specimen area was 1 cm^2^. This test was done with and without the extract of *OOL* as the bio-inhibitor in an optimum concentration. For Tafel polarization tests, a DC voltage was applied with the amplitude of ±250 V around the OCP. This potential was scanned at a speed of 0.1 mV s^−1^. To show the inhibition efficiency (*θ* × 100) the relative difference of corrosion rate (*i*_corr_) of samples with and without bio-inhibitor was considered based on the Tafel polarization data [3, 27]. For EIS tests, an AC voltage with the amplitude of 5 mV was applied and the frequency range was 100 kHz to 10 mHz. In this test, the relative difference of polarization resistance (*R*_p_) of samples with and without bio-inhibitor was expressed as the inhibition efficiency [8]. EIS data was fitted through a proposed electrical circuit. Besides, a Zview software was used to measure the value of electrical elements in the circuit. For both electrochemical tests, about three specimens in each situation were analyzed to show the repeatability of the work.

In addition to electrochemical tests, the gravimetric test was also utilized. For this test, at first, samples were weighted by the accuracy of ±0.1 mg. Then, they were immersed in the corrosive solution with and without bio-inhibitor at various temperatures and inhibitor concentrations. After specific times, samples were dried. Corrosion products from the surface were cleaned up by fine brushing and rubbing acetone on the surface of the specimens. At last, specimens were weighed again. Therefore, the weight loss was reported versus the immersed time for samples. To report the inhibition efficiency (θ × 100) the relative difference of weigh loss of samples with and without bio-inhibitor was utilized [4, 5].

It was noticeable that for thermodynamic and kinetic measurements the following equations were used, respectively [8, 9].

(1) $$\Delta G_{ads}^{{}^{\circ}}=-RT\;\ln\left(1000K_{ads}\right)$$

In Eq. (1), $\Delta G_{ads}^{{}^{\circ}}$ is the standard free energy when the utilized inhibitor molecular adsorbed on the steel substrate. *R* was the gas constant and *T* is the solution temperature. Besides, $K_{ads}$ is the constant of the adsorption process in the equilibrium condition [8,11].

(2) $$i_{corr}=A\;\exp\left(\frac{-E_{a}^{*}}{RT}\right)$$

In Eq. (2), $E_{a}^{*}$ is the activation energy of corrosion reactions for steel substrates.

(3) $$i_{corr}=\frac{RT}{Nh}\exp\left(\frac{\Delta S^{*}}{R}\right)\exp\left(\frac{-\Delta H^{*}}{RT}\right)$$

In equation (3), N and h are the *Avogadro*'s number and the *Planck*'s constant, respectively. $\Delta S^{*}$ is the entropy and $\Delta H^{*}$ is the enthalpy for corrosion reactions of steel substrates in the corrosive solution.

For the corroded surface evaluation, both AFM and FESEM images of samples with and without the extract of *OOL* as the inhibitor at the optimum concentration were studied. Samples were exposed to the corrosive solution for 1 and 72 h for AFM and FESEM investigation, respectively. For measurement of surface roughness through AFM micrographs, FemtoScan equipment was used. FESEM images were achieved through a TeScan Mira 3 apparatus equipped with the EDS method.

To study the chemical composition of corrosion products both GIXRD and FTIR- ATR methods were performed. The FTIR- ATR analysis was also carried out with a model Bruker-Equinox 55- equipment. The wavenumber range was from 600 to 4000 cm^−1^ with a step size of 1 cm^−1^. Samples with the corroded surface after 72 h without and with the inhibitor was utilized for this test. A Malvern PANalytical GIXRD equipment was used to detect formed phases at the corroded surface of steel after 72 h immersion in the acidic solution. The range of 2*θ* was 5–80^◦^ when a step size of 0.02^◦^ was applied.

## 3 Results and discussion

### 3.1 FTIR study

**Figure 2:**
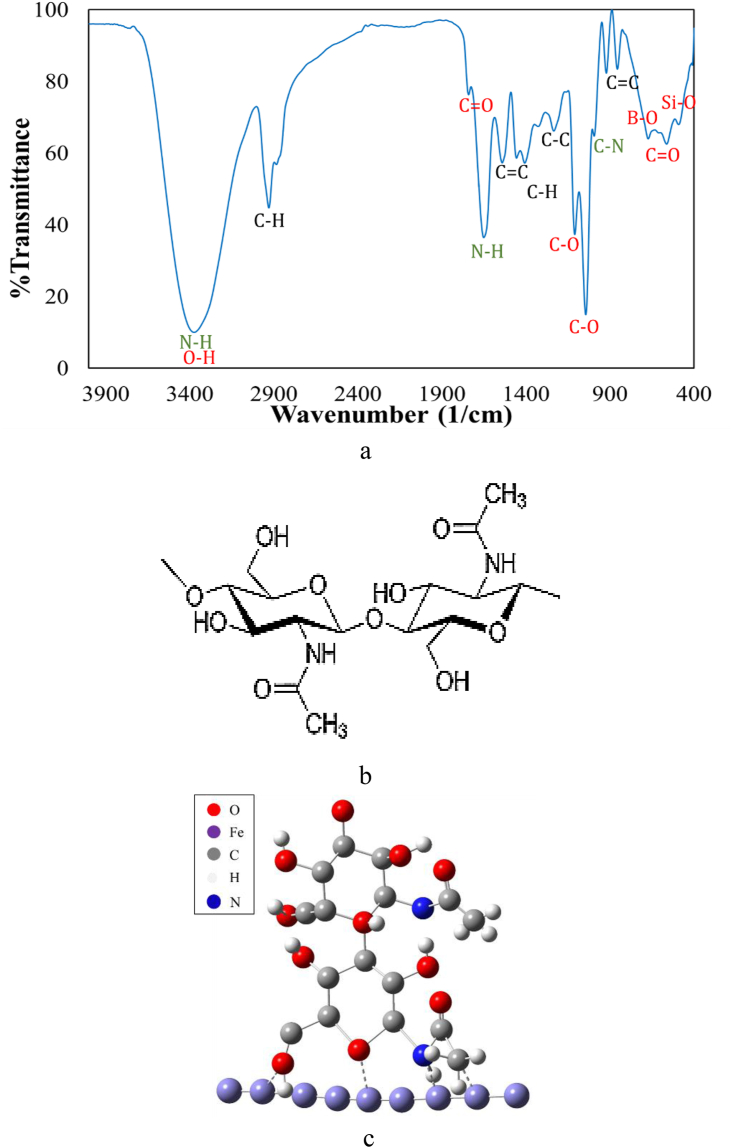
(a) The FTIR result for the extract of *OO*L (b) the schematic structure of chitin, and (c) the simulated image of the proposed adsorption mechanism of inhibitor molecular.

Figure 2(a) shows the FTIR pattern for the extract of *OOL*. The dominant broad peak was related to the N–H or O–H bond at the wavenumber of 3374 cm^−1^. The N–H bonding showed a peak at the 1651 cm^−1^. Besides, a peak at 1741 cm^−1^was related to the C=O bond. A C–N bond had a peak at the wavenumber of 994 cm^−1^. Totally, these four peaks showed that the amide group 2 (or protein) would be present in the *OO*L extract. A similar observation was also found by Bidi et al. [8]. It was notable that the C=O bending bond demonstrated a peak at 563 cm^−1^. In addition, another dominant sharp peak was at the wavenumber of 1044.1 cm^−1^ and has corresponded to the C–O bond. Besides, this bond showed another peak at 1109.6 cm^−1^. Peaks at 2929 and 1456 cm^−1^ were related to the C–H bending bond. It was indicated that the C–H stretching bond in aromatic alkane groups showed similar peaks [9]. Peaks of 1541 and 921 were related to the C=C bond in aromatic and aliphatic cyclic structures, respectively. The C–C bond at the cyclic structure demonstrated a peak at 1234 cm^−1^. The Si–O and B–O (in the BO_3_ compound) bonds had peaks at 478 and 672 cm^−1^.

It was found that the body of *OO*L has consisted of chitin (the most part), protein, and peptides [14]. Chitin (C_8_H_13_O_5_N)_n_, a derivative of glucose, is a long-chain polymer of N-acetylglucosamine. The monomer of chitin is shown in Figure 2(b). Figure 2(c) displays the simulated image of the proposed adsorption mechanism of chitin molecular. The adsorption could be done through the unboned electron pairs of O and N atoms. Since Fe atoms had vacant d-orbital. In addition, C atoms with a positive charge could adsorb by Fe atoms of surface since the Fermi level of electrons for metals was high [17]. It was notable that the simulated image in Figure 2(c) was prepared through Gauss view software.

### 3.2 Tafel polarization at 301 K

**Figure 3:**
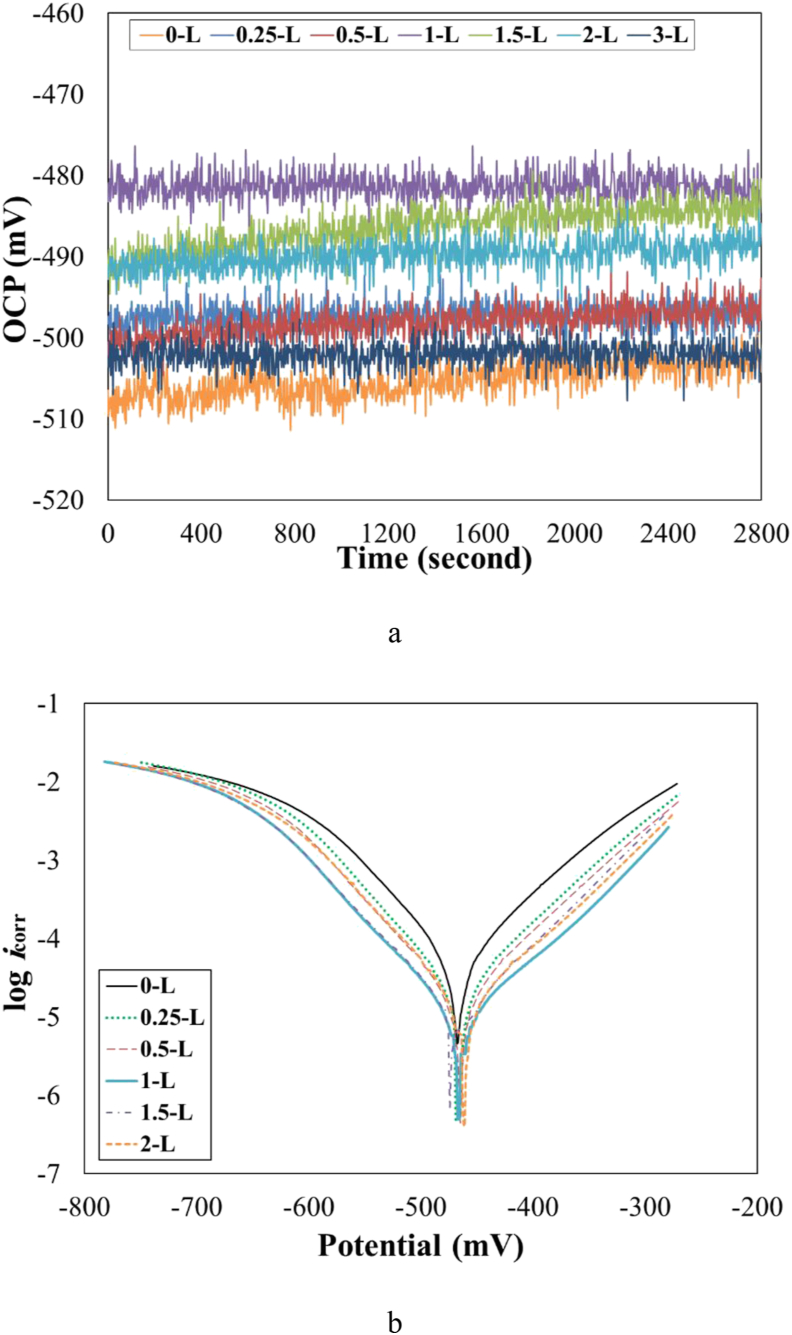
(a) Plots of OCP versus the exposure time, and (b) The Tafel polarization plots for various samples at a temperature of 301 K.

Figure 3(a) shows plots of OCP versus the exposure time for various samples at a temperature of 301 K. In all plots, at the initial time, the OCP value changed toward the positive value slightly, and by passing more time, it remained at a steady value. This trend was also reported by Lai et al. [18]. However, the reverse trend was found in other research [8]. The OCP value for steel substrate was more negative without the inhibitor in the acidic solution. This value was about –505 mV. When the *OO*L extract added to 1 M HCl solution OCP values increased. The highest OCP with a value of –480 mV was related to the specimen 1–L. It was found that more negative voltage resulted in an increased tendency for the anodic reaction from a thermodynamic point of view [8]. Therefore, the lowest tendency to Fe dissolution was achieved when the inhibitor concentration was 1 g/L. In addition, changes in OCP values could indicate that the adsorption of *OOL* extract at the carbon steel surface would have happened. A similar trend was also reported by other research [4].

Figure 3(b) displays the Tafel polarization plots for various samples at a temperature of 301 K. Besides, the extracted data from these plots are reported in Table 3. By adding the utilized inhibitor in various concentrations the corrosion rate or *i*_corr_ for the steel substrate decreased significantly. The decrease in the value of *i*_corr_ would be related to the adsorption of inhibitor compounds, through oxygen and nitrogen atoms and π-bonds in its functional group (*i.e*. O–H, C–OH, C=O, N–H) toward the metallic surface. It was noticeable that the aromatic rings could also block the active sites on the surface of metals by the formation of an insulating layer. A similar observation was also reported by Varvara et al. [19].

**Table 3:** The Tafel polarization data for various samples at a temperature of 301 K.

sample name	i_corr_ (Acm^−2^)	β_a_ (V/dec)	β_c_ (V/dec)	E_corr_ (mV)	θ
0–L	0.0681 ± 0.01	0.0713 ± 0.02	–0.1076 ± 0.02	–482.2 ± 1.1	–
0.25–L	0.0258 ± 0.01	0.0635 ± 0.02	–0.0982 ± 0.02	–479.6 ± 1.1	0.62
0.5–L	0.0208 ± 0.01	0.0651 ± 0.02	–0.0987 ± 0.02	–481.1 ± 1.1	0.69
1–L	0.0094 ± 0.01	0.0607 ± 0.02	–0.0973 ± 0.02	–475.1 ± 1.1	0.86
1.5–L	0.0109 ± 0.01	0.0588 ± 0.02	–0.0983 ± 0.02	–474.4 ± 1.1	0.84
2–L	0.0155 ± 0.01	0.0633 ± 0.02	–0.1035 ± 0.02	–479.1 ± 1.1	0.77
3–L	0.02899 ± 0.01	0.2870 ± 0.02	–0.3509 ± 0.02	–490.0 ± 1.1	0.57

The results in Table 3 show that the lowest *i*_corr_ value was obtained in the solution containing 1 g/L of the extract of *OO*L. By increasing the inhibitor concentration higher than this value, the *i*_corr_ values would be increased. Accordingly, the efficiency of the inhibitor was the highest (with a value of 86%) in the presence of l g/L of the *OO*L extract. In all, the inhibition efficiency was about 57–86%. When the concentration of inhibitor was less than the optimum content, the concentration was not enough to form a protective layer; however, by increasing the inhibitor concentration to 3 g/L, in the protective layer, some cavities would be created to permit the diffusion of corrosive ions toward the steel substrate and caused an increase in the corrosion rate. A similar trend was also reported by another researcher [19]. It was noticeable that the inhibiting efficiency was a parameter that indicating the ability of an organic compound to adsorb on the surface [20]. It was reported that by increasing the concentration of the inhibitor to 3 g/L, the inhibition efficiency increased [13]. However, such an event was not observed in this study.

No obvious trend has been observed in the shifting of *E*_corr_ values at different concentrations of bio-inhibitor. However, since these changes were lower than 85 mV, the bio-inhibitor acted as a mixed inhibitor. This was also found by Ghelichkhah et al. [21]. The range of *E*_corr_ for all samples was from –490 to –474 mV. In this situation, it was reported that a geometric blocking effect of inhibitor molecules would be achieved [11]. The slope of the anodic branch was slightly lower than the cathodic branch. It was found that in this situation, the anodic depolarization process was controlled by the adsorption of inhibitor molecules with high electrochemical stability [16]. Thus, the anodic reaction (Fe = Fe^2+^ + 2 e^−^) was affected lower than the cathodic reaction (H^+^ + 2 e^−^ = H_2_) in presence of the *OO*L extract at a temperature of 301 K. Values of both *β*_a_ and *β*_c_ were in good agreement with results reported previously for iron in 1 M HCl solution [4].

### 3.3 EIS at 301 K

**Figure 4:**
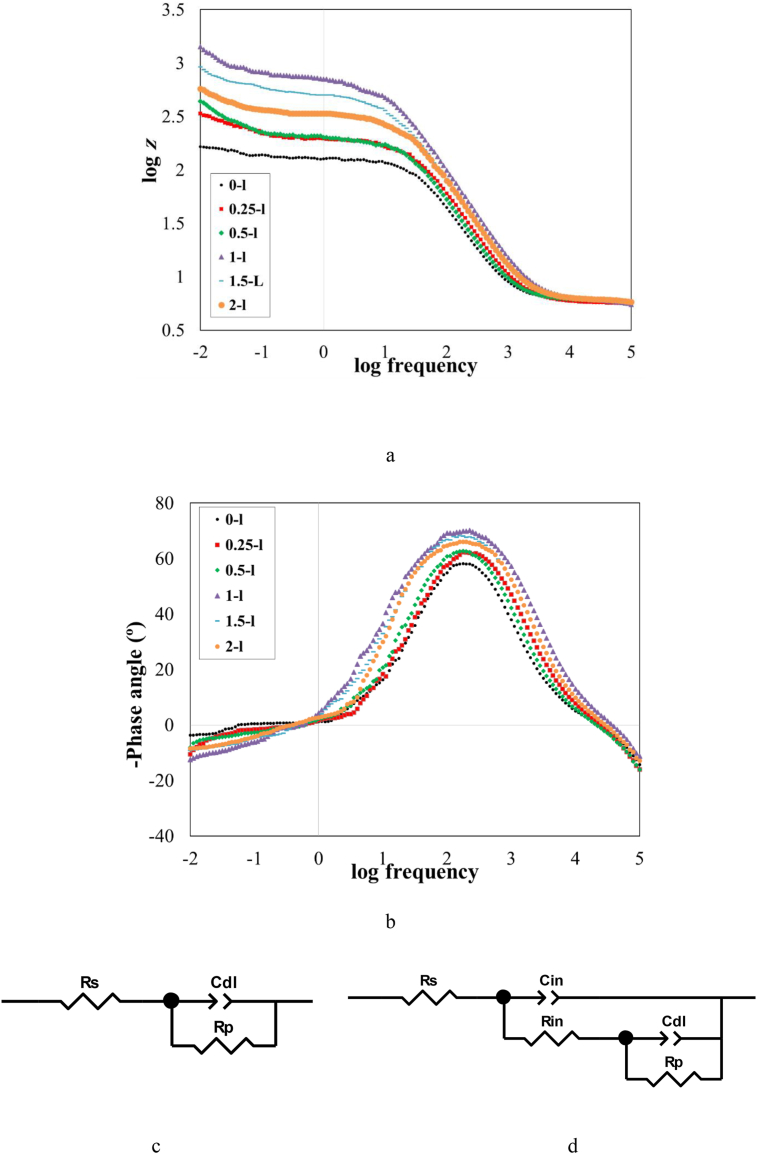
(a) Bode plots, and (b) phase-angle plots for various samples at a temperature of 301 K, (c) One-time and (d) two-time constant proposed electrical circuit fitted for EIS plots.

Figure 4 displays Bode and phase-angle plots for various samples at a temperature of 301 K. Moreover, Nyquist plots are presented in Supplementary (S1). As shown in Figure 4(a), when the extract of *OO*L was included in the acidic solution, the impedance modules increased significantly. In addition, based on the phase-angle plot shown in Figure 4(b), the angle decreased significantly from –48 to –73° with the utilized bio-inhibitor. Both of these plots indicated that the presence of the utilized bio-inhibitor in the 1 M HCl solution increased the corrosion resistance of steel corrosion. It was reported for an ideal surface with no roughness the phase angle would be –90° [22,23]. Consequently, when the acidic solution contained 1 g/L of *OO*L extract, the highest phase angle was achieved. This was suggested that by adsorbing the inhibitor molecules, the roughness of the surface reduced obviously. Moreover, a similar shape of Bode plots was achieved when the inhibitor concentration was 0.5, 1, and 1.5 M; however, other samples showed a different feature. It was found that the similar Bode plots showed a similar mechanism for the corrosion process [16].

To investigate more detail through the Zview software EIS data were fitted to equivalent electrical circuits. The proposed electrical circuits are displayed in Figure 4(c) and (d). As shown in Figure 4(c), in a one-time constant electrical circuit, *R*_p_ and *R*_s_ were the polarization and the solution resistance, respectively. The C_dl_ was the constant phase element of the double layer. This circuit was proper for samples of 0–L, 0.25–L, and, 2–L. A similar propose model for the electrical circuit was also suggested by other research [22, 24]. It was notable that the capacity of the double layer replaced by the constant phase element due to the roughness of the surface. The roughness of the surface resulted in the deviation from the ideal behavior of the capacitor. This was the result of various parameters such as adsorption of corrosive ions and molecules of inhibitor at the surface, corrosion reactions on the surface, the non-uniformity of corrosion products, and the complex chemical composition of the created layer on the surface [4, 16]. In Figure 4(d), *C*_in_ and *R*_in_ were the constant phase element and resistance of the layer formed on the steel substrate in the two-time constant electrical circuit. This layer had a complex composition that contained corrosion products and inhibitor molecules. It was notable that since the corrosion product was porous the double layer created on the steel surface. This circuit was proper when the concentration of bio-inhibitor was 0.5, 1, and 1.5 M. A similar model for the equivalent electrical circuit was also proposed by other research [25].

Table 4 shows the extracted data from the EIS test results. The change in the value of *R*_s_ with or without the inhibitor was insignificant indicating this resistance had no effective role in the inhibition mechanism. The value of *R*_s_ was about 6 Ω for all samples. This could be also observed from the Bode plots (the impedance magnitude at the frequency of 100 kHz). By the presence of the extract of *OOL* in the acidic solution, *R*_p_ increased significantly due to the adsorption of inhibitor molecules on the surface. When inhibitor concentration increased from 0.25 to 1 g/L, the *R*_p_ increased; however, by adding more inhibitor to 1 g/L, *R*_p_ decreased. Totally, the range of inhibition efficiency was about 50–83%. The highest value of *R*_p_ was related to the sample 1–L, when the inhibitor concentration was about 1 g/L. This result was consistent with the Tafel polarization data. In addition, when inhibitor concentration was from 0.5 to 1.5 g/L, a stable layer of inhibitor molecules formed on the steel substrate to create the second loop in the electrical circuits. The highest value of *R*_in_ has also corresponded to the sample 1–L. This was demonstrated that although a layer was formed through the adsorption of inhibitor molecules at the surface, it could not make effective protection to block all corrosive species in higher concentrations of inhibitor. In other words, in an optimum concentration of inhibitor (with a value of 1 g/L), the layer formed on the surface played an effective role to decrease the rate of corrosive ion migration toward the surface; however, when more inhibitor was involved, new cavities could be produced in the layer. This caused a reduction in the protective ability of the layer. In this situation, to achieve the highest protection, the inhibitor concentration should be carefully selected.

**Table 4:** Extracted EIS data for various samples.

sample name	R_s_ (Ω)	C_dl_ (μFcm^−2^)	n	R_p_ (Ωcm^2^)	θ	R_in_ (Ωcm^2^)	C_in_ (μFcm^−2^)	n
0–L	6.0	60.5	0.81	120.9	–	–	–	–
0.25–L	5.7	44.9	0.90	239.1	0.50	–	–	–
0.5–L	6.0	52.8	0.91	257.4	0.53	152.1	132.1	0.71
1–L	5.9	32.6	0.89	730.2	0.83	356.1	102.4	0.75
1.5–L	6.1	36.5	0.90	557.5	0.79	231.1	124.6	0.68
2–L	5.8	51.2	0.88	436.9	0.72	–	–	–

By adding the extract of *OO*L, *C*_dl_ decreased. This value could be related to the changes in the adsorbed species in presence of molecules of the *OO*L extract. In this situation, a reduction in the dielectric constant would have occurred. It was found that by replacing water molecules with other ions and molecules and changes in the thickness of formed film on the surface, the capacity of the double layer would be changed [11,22]. Moreover, the lowest values of *C*_dl_ and *C*_*i*n_ has corresponded to the sample 1–L. The value of *n* for all specimens was about 0.81–0.91.

### 3.4 Modeling of the adsorption mechanism

**Figure 5:**
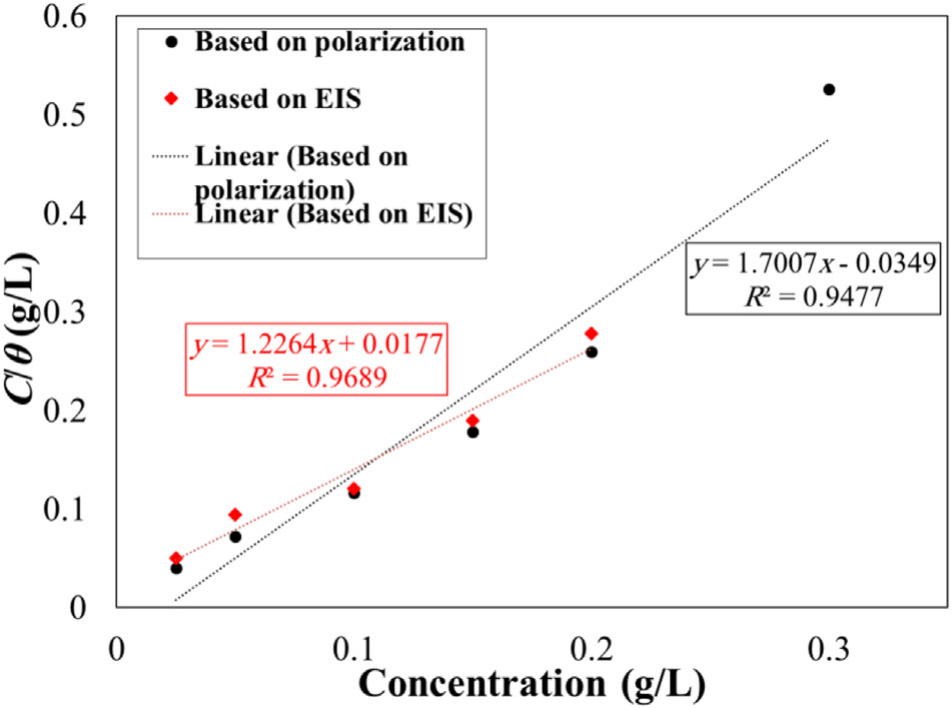
Plots of *C*/*θ* versus the inhibitor concentration based on Tafel polarization and EIS data.

To explore the adsorption mechanism of the utilized inhibitor on the surface, various possible adsorption models were studied based on the experimental data. These models contained Freundlich, Langmuir, Langmuir-Freundlich, Flowery-Huggins, Temkin, and Frumkin [11, 16, 22]. As shown in Figure 5, due to the value of the correlation coefficient (*R*^2^), the proposed adsorption mechanism for molecules of the *OO*L extract was the Langmuir adsorption model. It was reported that when the slope of the plot (*C*/*θ* versus the inhibitor concentration) was unit, the proposed model of adsorption was the Langmuir isotherms [16]. In this model, monolayer adsorption of molecules of the *OOL* extract had happened at the steel surface and there was no lateral interaction would be occurred between them [26].

Moreover, the reverse of the plot slope was proportional to the constant of equilibrium in the adsorption process (*K*_ad_) [22]. Therefore, based on both electrochemical test data, values of calculated *K*_ad_ were reported, as shown in Table 5. Besides, through Eq. (1), values of Δ*G*º_ads_ were also calculated and reported in Table 5. The negative value of Δ*G*º_ads_ showed that the adsorption process for molecules of the extract of *OO*L at the steel surface was spontaneous, however, the value of this thermodynamic parameter was not high and was about –16 kJ/mol. This event showed the type of adsorption obeyed from a physical model. In this situation, an electrostatic bond between the inhibitor molecular and steel surface has happened. It was indicated that when the value of Δ*G*º_ad_ was lower than –20 kJ/mol, the physisorption of molecular at the surface was predominant [21].

**Table 5:** Calculated values of *K*_ad_ and Δ*G*º_ads_ at 301K.

Calculations based on Tafel polarization data	
*K*_ad_ (L/g)	Δ*G*º_ads_ (kJ/mol)
0.588	–15.96
Calculations based on EIS data	
0.815	–16.78

### 3.5 Gravimetric investigation at 301 K

**Figure 6:**
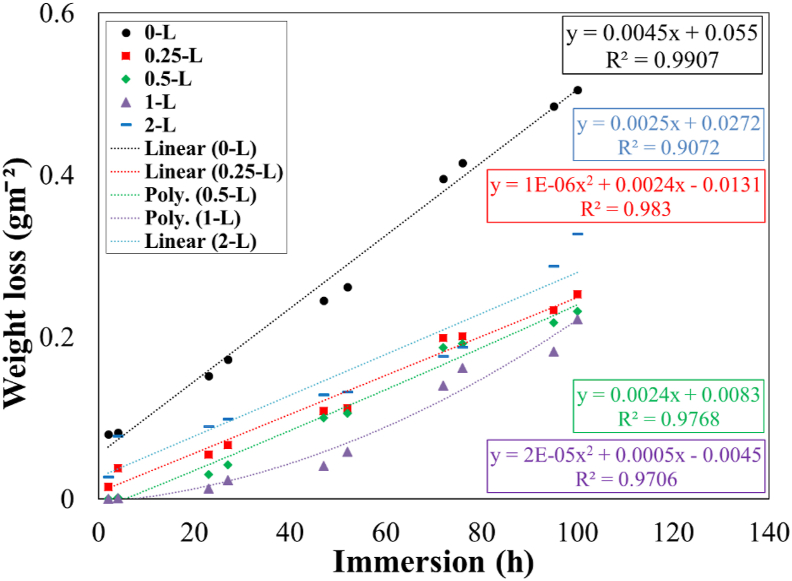
Plots of weight loss versus the immersion time in 1 M HCl solution at 301 K for various specimens.

To study the effect of long immersion time (up to 100 h) on the corrosion behavior of steel substrate, the weight loss tests were performed for various samples. Based on the results of electrochemical tests, four concentrations of the *OO*L extract contained 0.25, 0.5, 1, and 2 g/L were chosen for this test. The dissolution behavior of Fe atoms with and without the *OO*L extract in the acidic solution at 301 K is shown in Figure 6. Besides, the extracted data of plots in Figure 6, are reported in Table 6.

**Table 6:** Extracted data from weight loss test results.

	Immersion time (h)										
sample name	2	4	23	27	47	52	72	76	95	100	*θ*_ave_
0.25–L	0.81	0.54	0.64	0.61	0.56	0.57	0.50	0.52	0.52	0.50	0.58
0.5–L	1	0.98	0.80	0.76	0.59	0.60	0.53	0.54	0.55	0.54	0.69
1–L	1	0.99	0.91	0.87	0.83	0.78	0.65	0.61	0.62	0.56	0.78
2–L	0.66	0.56	0.41	0.43	0.48	0.50	0.55	0.55	0.41	0.35	0.44

Due to the slope of plots, it was indicated that the corrosion rate of steel substrate was high without the inhibitor; however, when the extract of the *OO*L added to the acidic solution, corrosion rates decreased significantly. The highest mean value of *θ* was about 0.75 over the entire time of exposure time when the inhibitor concentration was about 1 g/L. By increasing the time exposure, the value of *θ* decreased. This was due to the decrease in the adsorption process of inhibitor molecules at the surface by elongating time. In other words, fast adsorption of inhibitor molecules has happened, and then by increasing the time, the reduction in the inhibition performance would have occurred. Besides, the presence of Cl^−^ ions at the surface caused the breakdown of inhibitor film continuity. This was also reported by Deivanayagam et al. [9].

Based on calculated equations in Figure 6, two models of weight loss versus the immersion time would be suggested as follows (equation (4(4) and (5)(5)):

(4) w=at+b

(5) $$w=at^{2}+bt-c$$

where *w* is the weight loss, *t* is the immersion time, *a*,*b*, and *c* were constants dependent on the concentration of inhibitor. Eq. (4) showed that the corrosion rate was linear over the immersion time; however, for Eq. (5) a non-linear property was observed. When the inhibitor concentration was about 0.5 and 1 g/L, the weight loss was obeyed from a non-linear rule. This was consistent with EIS data. In these two concentrations, a protective layer would be formed on the steel surface to reduce the corrosion rate significantly, however, the stability of this layer was affected by long immersion times and would be ruptured.

### 3.6 OCP and Tafel polarization at the range of 298–313 K

**Figure 7:**
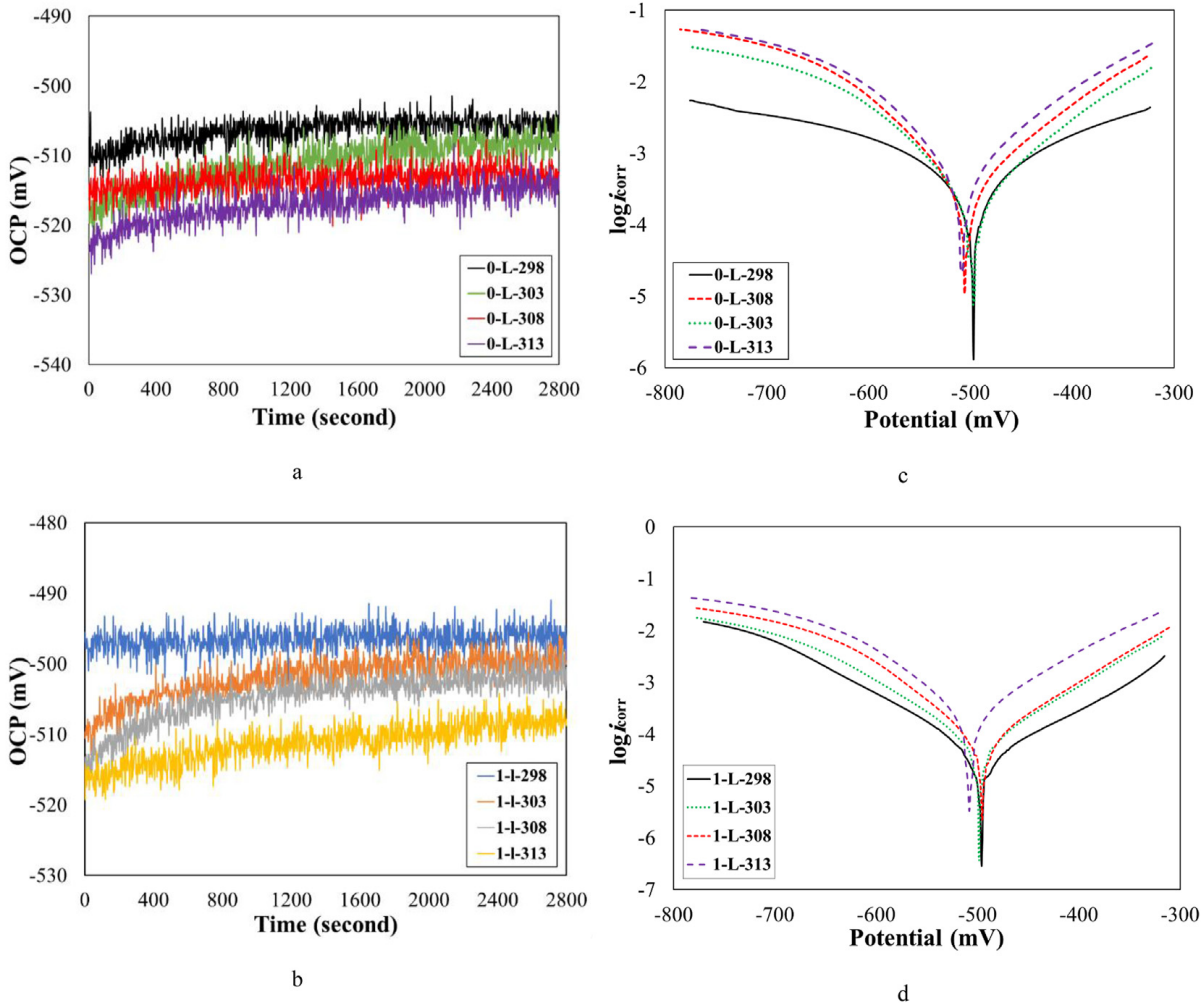
Plots of OCP versus the immersion time at various temperatures (a) without and (b) with the *OO*L at the concentration of 1 g/L, Tafel polarization test data at various temperatures (c) without and (d) with 1 g/L *OOL* as bio-inhibitor.

To investigate the influence of higher temperatures on the corrosion behavior of steel substrate in 1 M HCl solution, OCP measurements, and Tafel polarization test at the range of 298–313 K were utilized. Figure 7(a) and (b) demonstrate plots of OCP versus the immersion time at various temperatures without and with of the extract of *OOL* at the concentration of 1 g/L, respectively.

By increasing the temperature from 298 to 313 K, OCP values decreased and become more negative without and with 1 g/L inhibitor in the solution. This event showed that the tendency to the dissolution of Fe atoms increased at the higher temperature. In addition, the range of OCP values in the presence of *OO*L extract was lower in comparison with the other condition. This range was about –495 to –515 mV. The OCP range in absence of the utilized bio-inhibitor was about –505 to –525 mV. A similar behavior (a reduction in the OCP value with the temperature) was also found in another study [8].

**Figure 8:**
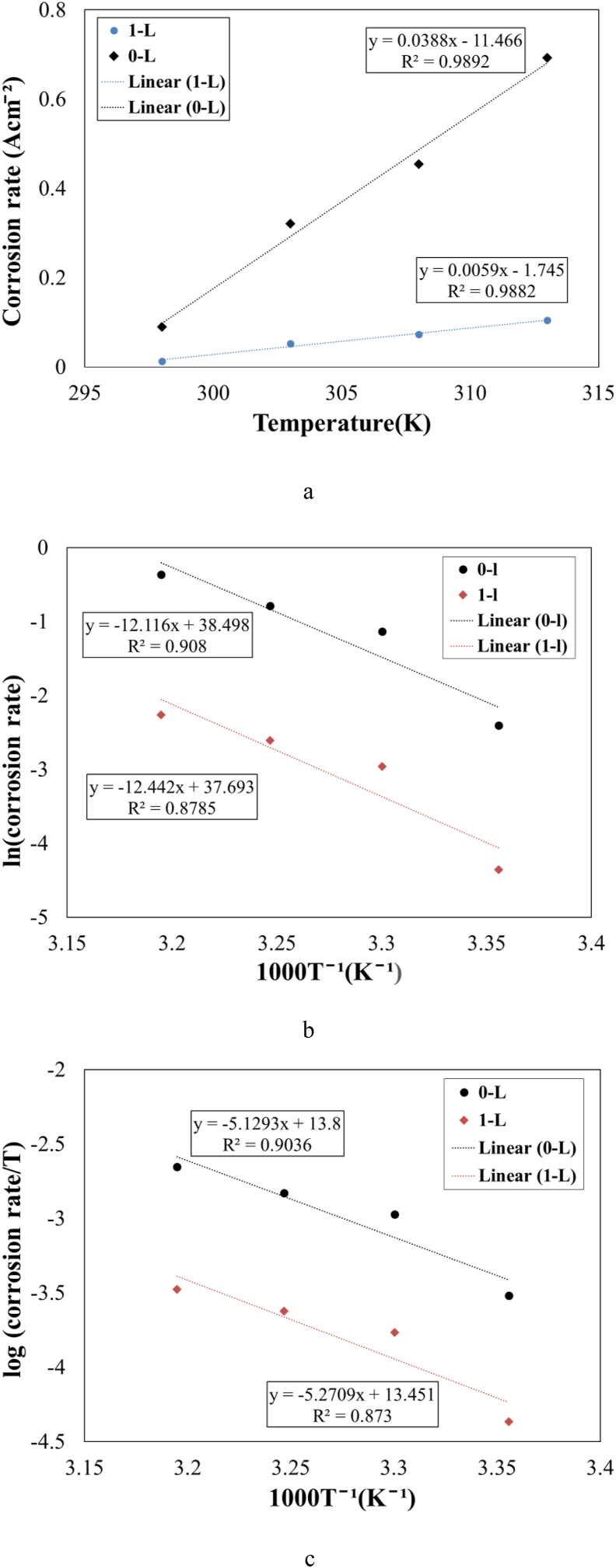
(a) Plots of *i*_corr_ versus temperature, (b) ln (*i*_corr_) versus temperature, (c) log (*i*_corr_/T) versus temperature for samples 0–L and 1–L.

Figure 7(c) and (d) show the Tafel polarization plots versus temperatures without and with 1 g/L *OOL* extract as bio-inhibitor. Besides, the extracted data from these plots are reported in Table 7. Similar to changes of OCP values by the temperature, by increasing the temperature, *E*_corr_ values decreased and went toward more negative potential. However, changes in these values were not significant. The corrosion rate (*i*_corr_) of steel substrates increased without and with 1 g/L *OOL* by increasing the solution temperature. The increased range of *i*_corr_ was about 71.9–86.9% and 75.3–87.7% without and with 1 g/L *OO*L, respectively. Moreover, changes in corrosion rates versus the temperature were higher when the inhibitor was not present in the solution, as shown in Figure 8(a). The slope of the plot (*i*_corr_ versus temperature) was about 0.0388 and 0.0059 Acm^−1^T^−1^ for samples of 0–L and 1–L, respectively. Moreover, both values of *β*_c_ and *β*_a_ in all temperatures were lower when the bio-inhibitor was present in the solution. The inhibition efficiency was not changed by the temperature. Thus, by increasing the solution temperatures the stability of film formation of inhibitor molecules was not affected in the temperature range of 298–313 K significantly.

**Table 7:** Tafel polarization test data at various temperatures without and with 1 g/L *OOL*.

Samples	i_corr_ (Acm^−1^)	β_a_ (V/dec)	β_c_ (V/dec)	E_corr_ (mV)	% Increase in i_corr_	θ
0–L–298	0.0900	0.0895	–0.1411	–505.1	–	–
0–L–303	0.3213	0.1035	–0.1512	–508.5	71.9	–
0–L–308	0.4547	0.0819	–0.1266	–518.6	80.2	–
0–L–313	0.6923	0.0877	–0.1333	–525.1	86.9	–
1–L–298	0.0128	0.0892	–0.1484	–495.9	–	0.85
1–L–303	0.0518	0.0702	–0.1152	–504.7	75.3	0.84
1–L–308	0.0734	0.0723	–0.1109	–507.2	82.6	0.84
1–L–313	0.1041	0.0870	–0.1220	–511.4	87.7	0.85

For the investigation of more details about kinetical parameters, both Eqs. (2) and (3) were utilized. Figure 8(b) and (c) display plots of ln *i*_corr_ and log (*i*_corr_/T) versus the range temperature of 298–313 K. Besides, the extracted parameters are reported in Table 8.

**Table 8:** Calculated data for *E*_a_∗, Δ*H*∗, and Δ*S*∗ for samples 0–L and 1–L.

Samples	E_a_∗(kJ/mol)	ΔH∗ (kJ/mol)	ΔS∗ (J/K.mol)
0–L	100.7	–98.2	66.6
1–L	103.4	–100.9	59.9

The higher value of *E*_a_∗ for the sample 1–L compared to the sample 0–L demonstrated that the activation energy for the corrosion reaction of steel with the inhibitor increased. This event resulted in a reduction in the corrosion rates of steel substrates when molecules of *OO*L extract were added to the acidic solution. Since the change in the activation energy was not significant with and without the inhibitor, it suggested that inhibitor molecules had a geometric blocking effect at the steel surface to hinder the diffusion of corrosive ions toward the surface [11].

The value of Δ*H*∗ for samples 0–L and 1–L was about –98.2 and –100.9 kJ/mol, respectively. The negative value of Δ*H*∗ showed that the corrosion process of steel substrate (dissolution atoms of Fe) with and without inhibitor molecules was followed by the exothermic reaction. It was found that when the Δ*H*∗ was negative and low, it could be suggested a physical adsorption model [24]. It was noticeable that the value of was *E*_a_∗ was slightly larger than the value of Δ*H*∗. This suggested that the corrosion reaction involved a gaseous reaction (the hydrogen evolution) that was related to a reduction in the reaction volume [27]. The value of *E*_a_∗– Δ*H*∗ for both samples was 2.5 kJ/mol that was equal to *RT* (≈2.5 kJ/mol). This event was related to a uni-molecular reaction.

In addition, values of Δ*S*∗ for samples 0–L and 1–L were about 66.6 and 59.9 J/K.mol, respectively. It was found that Δ*S*∗ was related to the disordering of reactant molecules when they changed to activated complexes [9]. In other words, the positive value of Δ*S*∗ indicated that the ordering of molecules was low. Thus, when the inhibitor was added to the solution the ordering of molecules decreased insignificantly compared to the samples 0–L. Similar behavior was also found by other research [11,24].

### 3.7 Gravimetric study at 313 K

**Figure 9:**
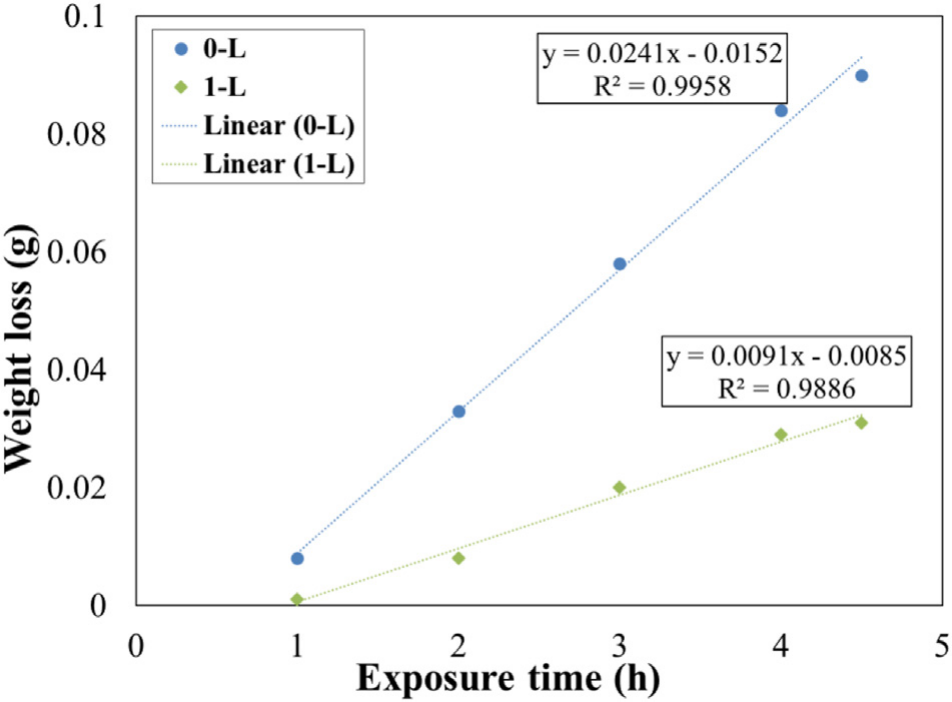
Plots of weight loss versus the immersion time in 1 M HCl solution at 301 K.

To study the effect of a higher temperature (313 K) versus the time, the weight loss test was performed and results are shown in Figure 9. When the exposure time increased up to 4.5 h, the weight loss of steel substrates increased with and without the extract of *OO*L at a concentration of 1 g/L in the solution. By increasing the time the inhibition efficiency decreased at a temperature of 313 K. The range of *θ* was about 0.87–0.65 over the time. It can be concluded that an increase in the temperature had no effect on the adsorption process; however, the exposure time played an effective role in the adsorption mechanism and a reduction in inhibition efficiency. It was noticeable that at a temperature of 313 K, the corrosion rate of carbon steel specimen with and without the extract of *OO*L was 0.0091 and 0.0241 g/h, respectively. Moreover, based on the value of *R*^2^, it seemed that the corrosion rate of steel versus the time at high temperature was obeyed from linear behavior.

### 3.8 FESEM images investigation

**Figure 10:**
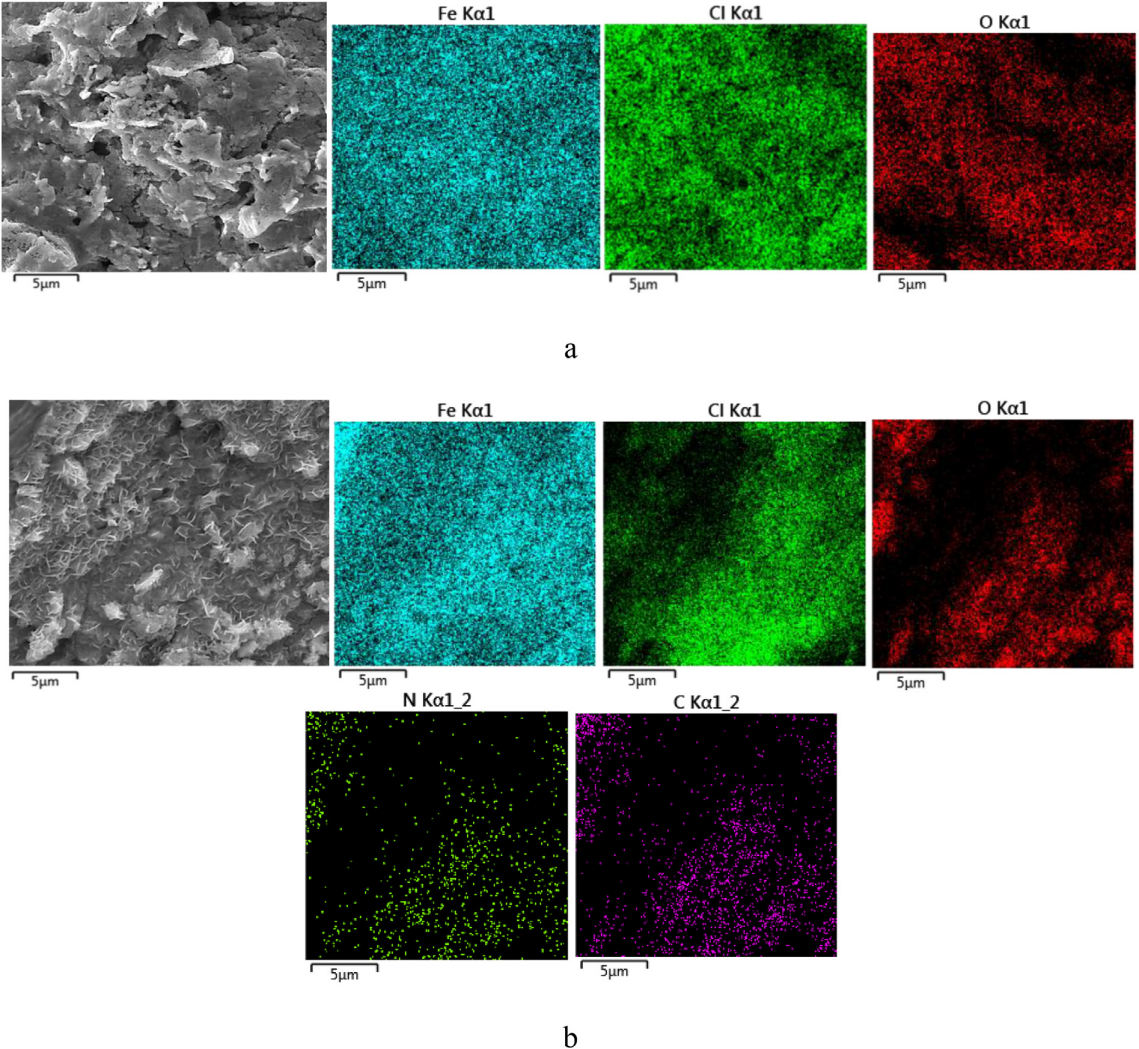
FESEM images and EDS map results of the corroded surface after 72 h immersion for (a) sample 0–Land (b) sample 1–L.

Figure 10(a) and (b) show the surface morphology of corroded surface without and with the bio-inhibitor, respectively, after immersion of 72 h. As shown in Figure 10(a), a high number of pits and cracks were created on the steel surface due to the corrosion reactions; however, when molecules of *OO*L extract at a concentration of 1 g/L adsorbed on the substrate the number of pits decreased significantly, as demonstrated in Figure 10(b). In addition, without the inhibitor, the oxide layer that formed on the surface was irregular. This layer had open pits to permit the easy access of corrosive ions to the substrate. However, a needle-shaped of iron oxide in a continuous-layer form was found for the sample 1–L. Besides, the corrosion products layer was more compact compared to the other sample. This event was consistent with the lower value of Δ*S*∗. A similar image was also reported by Antunes et al. [7]. Totally, when 1 g/L of inhibitor was added to the 1 M HCl solution, the morphology of the corroded surface changed obviously.

EDS method (in both map and dot modes) was applied to detect elements that were present on the steel surface with and without bio-inhibitor after immersion in the corrosive solution. The reported data in Table 9(a) show that when there was no inhibitor, the steel substrate was corroded in the acidic solution, due to the presence of O on the surface. Besides, the Cl^−^ ions adsorbed on the steel surface, since in the vicinity of the steel substrate, the Fe atoms changed to Fe^2+^ ions. When the optimum concentration of bio-inhibitor (g/L M) was added to the solution, other elements such as C and N were present on the corroded surface, as reported in Table 9(b). Moreover, in this situation, the content of the Cl^−^ ions decreased on the steel substrate when molecules of *OOL* extract adsorbed on the surface. The content of adsorbed Cl element on the corroded surface decreased from 16.84 to 13.77 wt% when the bio-inhibitor was added to the acidic solution. A similar reduction trend in the presence of adsorbed inhibitor molecules at the surface was also reported in other research [24]. Besides, in this case, the amount of adsorbed O element decreased from 9.05 to 6.98 wt%.

**Table 9a:** EDS result from the corroded surface after 72 h immersion for (a) sample 0–L and (b) sample 1–L.

Element	Wt%	A%
O	9.05	18.65
Cl	16.84	15.66
Mn	0.75	0.45
Fe	73.16	55.84

**Table 9b:** 

Element	Wt%	A%
C	3.96	13.07
N	0.38	1.07
O	6.98	17.28
Cl	13.77	15.41
Mn	0.54	0.39
Fe	73.79	52.79

### 3.9 AFM images evaluation

**Figure 11:**
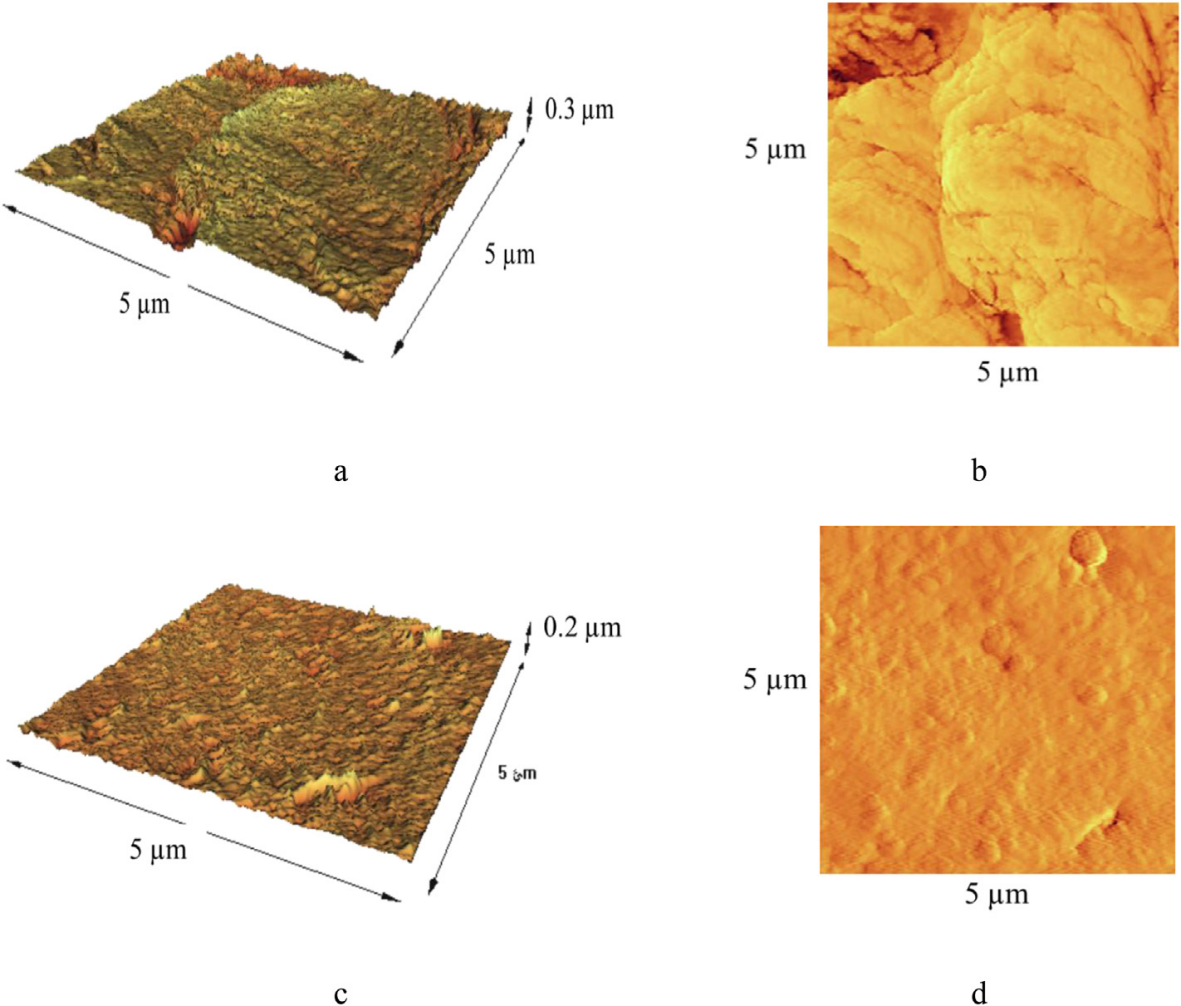
AFM images of corroded steel surfaces (a) and (b) without, (c) and (d) with 1 g/L *OOL* in the solution after 1 h immersion.

Figure 11 demonstrate AFM images of corroded steel surfaces after 1 h immersion in 1 M HCl solution without and with 1 g/L *OOL*.

As shown in Figure 11(a) and (b), without the inhibitor, the surface roughness of steel substrate was high and this was a result of the anodic dissolution reaction. When the corrosive solution contained 1 g/L *OO*L extract, the average roughness value reduced to 19.8 nm, as shown in Figure 11(c) and (d). This event showed that the intensity of corrosion reaction on the steel surface decreased in the presence of the *OO*L extract. This result was consistent with the value of Δ*S*∗ which showed that the ordering of species in the presence of the inhibitor increased. A similar result was also observed in other studies [8, 25].

### 3.10 GIXRD and FTIR-ATR investigations for corrosion products

**Figure 12:**
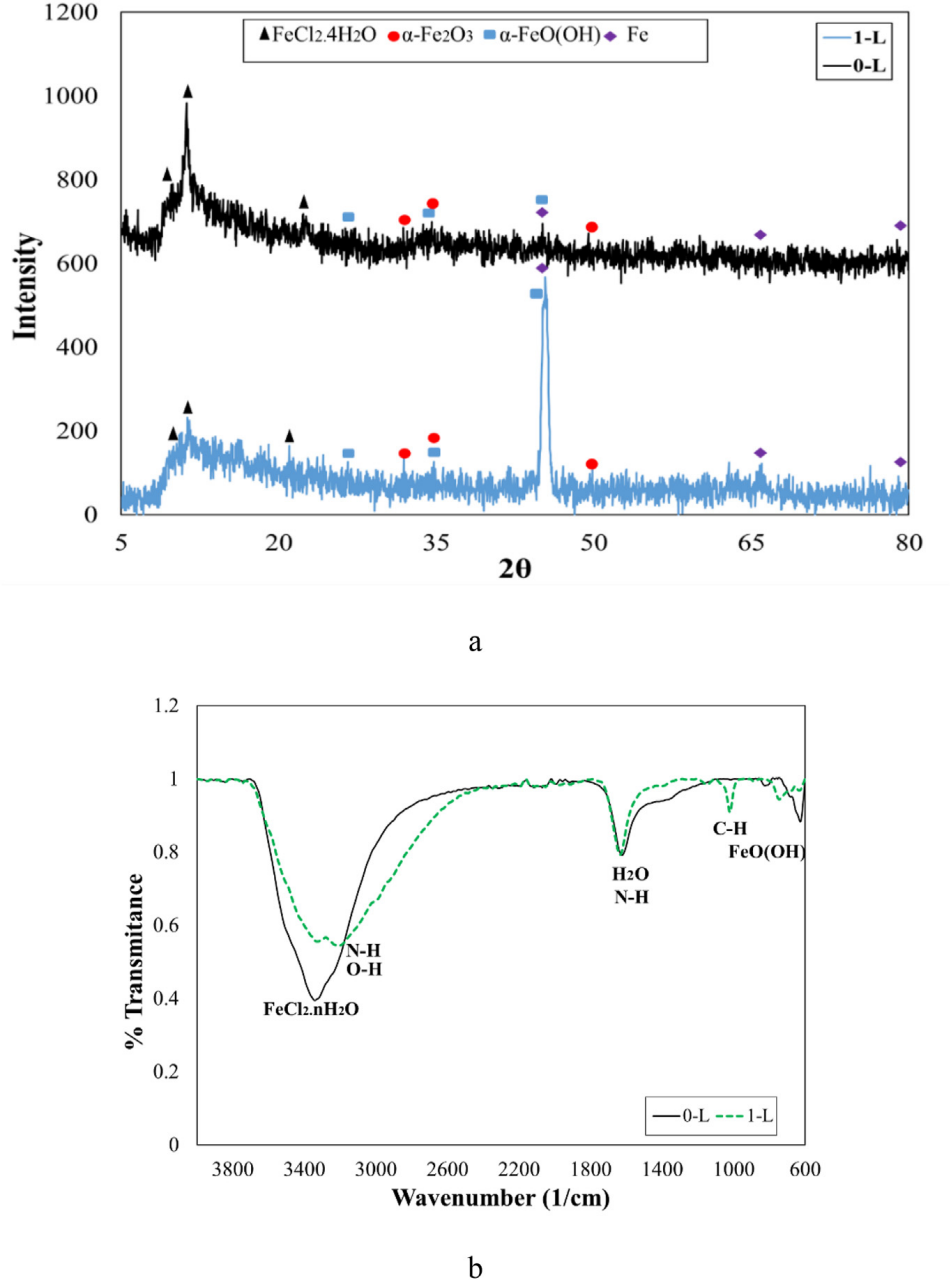
(a). GIXRD results and, (b) FTIR-ATR results from corroded surfaces after immersion of 72 h without and with 1 g/L *OOL* extract as the bio-inhibitor.

Figure 12(a) displays GIXRD patterns from corroded steel surfaces after immersion of 72 h without and with 1 g/L *OO*L extract. The highest peak for the sample 0–L was the FeCl_2_.4H_2_O phase as the corrosion product; however, other oxide phases such as α–FeOOH and α–Fe_2_O_3_ were found on the corroded surface. Therefore, the corrosion products were a mixture of chloride, hydroxide, and oxide phases of iron. It was reported that the surface of steel substrates after immersion in the acidic solution included various hydroxides, oxides, and other corrosion products [16]. In details, it was found that the corrosion products in HCl solution would be contained hydrated maghemite (γ–Fe_2_O_3_.H_2_O), goethite (α–FeOOH), FeO(OH, Cl), FeCl_2_, and FeCl_3_, FeO (OH, Cl) [6, 7, 28, 29].

When 1 g/L *OO*L extract was added to the solution, the GIXRD pattern changed insignificantly. The highest peak for the sample 1–L was related to the Fe phase from the substrate. This peak showed that the thickness of the corrosion product was low. Similar to results for the sample 0–L, the corrosion product of this sample contained FeCH_2_.4H_2_O α–FeOOH, and α–Fe_2_O_3_ phases. Moreover, for both samples, an amorphous phase of oxide iron was detected in an angle range of 7 to 15°. A similar result was also reported by other research [30].

Figure 12(b) demonstrates FTIR-ATR patterns from corroded steel surfaces after immersion of 72 h without and with 1 g/L *OO*L extract in 1 M HCl solution. When the solution did not contain any inhibitor, the dominant peak at 3341 cm^−1^ was related to the FeCl_2_. nH_2_O bond. This result was consistent with the GIXRD data, however, bonds for H_2_O and FeO(OH) were also detected at 1624 and 655 cm^−1^, respectively. In this situation, the water uptake at the steel surface has happened. When the acidic solution contained 1 g/L *OO*L extract, two other peaks at 3207 and 1019 cm^−1^ were added to the FTIR-ATR pattern. These were related to N–H and C–H bonds, respectively. This event confirmed the adsorption of *OO*L extract molecules at the surface. The peak intensity for iron chloride was lower for the sample 1–L compared to the sample 0–L. This could show the less corroded steel surface in the presence of the utilized bio-inhibitor. Similar to results for the sample 0–L, a peak at 1633 cm^−1^ showed that the molecules of water adsorbed on the surface and the hydrated corrosion products would be created on the final surface. Besides, this peak could be attributed to the N–H bond. Thus, the high intensity of this peak was a result of overlaying two near peaks to each other. This event indicated the replacement of small water molecules by large inhibitor molecules through the following reaction [11, 18];

[*OO*L]_sol_ + x [H_2_O]_ads_ = [*OO*L] _ads_*+* x [H_2_O] _sol_

The peak position for the FeO(OH) bond changed insignificantly from 655 to 743 cm^−1^ when the 1 g/L *OO*L extract added to the acidic solution. It was found that the peak displacement would have occurred in the presence of the inhibitor molecules that adsorbed on the steel surface [31]. Moreover, a peak at the wavenumber of 1750 cm^−1^ that was related to the hydronium ion (H_3_O^+^), was not found in ATR–FTIR patterns for both samples [32]. Since in aqueous acidic solutions, there was a tendency for molecules to be protonated [22].

### 3.11 Proposed inhibition mechanism

**Figure 13:**
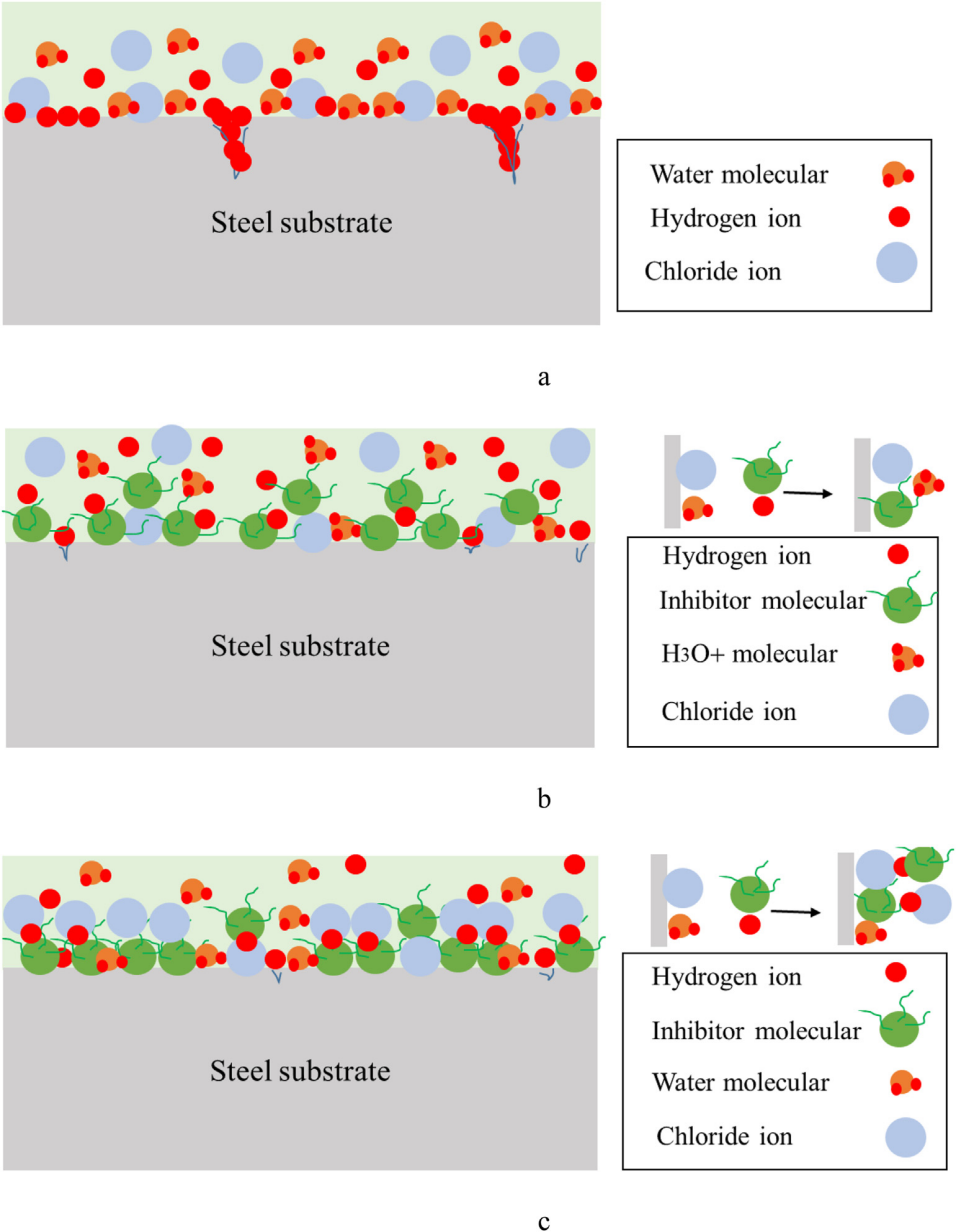
Proposed adsorption mechanism of molecules for (a) without, and (b) and (c) with the inhibitor in 1 M HCl solution.

Based on the obtained results, the suggested adsorption mechanisms are presented schematically in Figure 13. As shown in Figure 13(a), in the HCl solution, for the steel substrates corrosion reactions would have occurred. Therefore, due to the adsorption of H^+^ ions at the metallic surface, the dissolution of Fe atoms (Fe = Fe^2+^ +2 e^−^) would be done. In addition, since the Cl^−^ anions were adsorbed on the steel surface, the corrosion product of FeCl_2_ would be formed on the surface. Moreover, the uptake of water molecules has also happened at the steel surface. Thus, due to the result in Figure 12, the hydrated-FeCl_2_ would be the predominant corrosion products as FeCl_2_.4H_2_O. Plus this product, the iron oxide in small amounts was observed at the surface. In details, the dissolution rate of Fe was controlled by the following reactions [20].

Fe. H_2_O + Cl^−^ = [FeClOH]_ads_^−^ + H^+^ +e^−^

[FeClOH]_ads_^−^ = FeClOH + e^−^

FeClOH + H^+^ = Fe^2+^ + Cl^−^ + H_2_O

It was notable that in HCl solution, the metallic surface was positively charged and in this case, the Cl^−^ ions could be adsorbed at the surface [17, 33].

When the extract of *OOL* was added to the acidic solution, molecules of inhibitor were adsorbed on the carbon steel surface. This adsorption was a spontaneous process, based on data in Table 5. Polar groups of N and O in the organic compounds of *OO*L extract molecules was the center for the adsorption process. In this situation, two models of adsorption would be proposed [34, 35].

As displayed in Figure 13(b), the Cl^−^ anions plus the molecules of the *OO*L extract adsorbed separately at the steel surface and there was a competition between the adsorption of inhibitor molecules and H^+^ ions. When inhibitor molecules were replaced by water molecules at the steel interface, then, hydronium ion (H_3_O^+^) would be formed. In this condition, the adsorption of water molecules at the steel surface would have not occurred. Based on the results in Figure 12, this model was not dominant in this study.

As shown in Figure 13(c), the steel/*OOL* extract -Cl interfaces would be achieved. In this model, a co-operative interaction between the Cl^−^ anions and the molecules of the *OOL* extract would have occurred. In one mode, at first, Cl^−^ anions adsorbed at the surface and then the inhibitor molecules got protonated and could be adsorbed by Cl^−^ anions through the anodic site. In the other mode, the inhibitor molecules adsorbed from the side of N or O by the vacant d-orbital of Fe atoms. Then, the protonated molecules of inhibitor adsorbed the Cl^−^ anions. In this condition, the adsorption of inhibitor molecules acted as a physical barrier layer for H^+^ ions as the disrupting factor for the cathodic reaction. As shown in Figure 13(c), in this model, water molecules at the steel interface also adsorbed. Therefore, based on the results in Figure 12, this model would be suggested in this study. Thus, based on the Tafel polarization data, a physical barrier would be formed by the mixture of molecules at the interface to reduce the hydrogen evolution at cathodic sites. A similar mechanism was also proposed by other research [13,17,36].

However, by increasing the immersion time or inhibitor concentration, the stability of adsorption would be decreased or cavities in the formed layer on the steel substrate were created. In this situation, the corrosion rate increased. In addition, molecules of *OO*L extract could be broken into small fragments or reacted with other species in the solution. It was found that the molecular tail of chitin would be dissolved in acid, alkaline solution, and in the expose of high temperature. In this situation, the molecules of chitin would be changed to the chitosan structure [25].

## 4 Conclusion

The extract of *Oestrus ovis* larvae (*OO*L) as a potential and affordable bio-inhibitor was utilized in various concentrations to reduce the corrosion rate for carbon steel in the 1 M HCl solution. Various tests were performed to study this corrosion behavior. The Tafel polarization data at 301 K indicated that by adding 0.25–3 g/L of bio-inhibitor, the inhibition efficiency was about 57–86%. The optimum concentration of the *OO*L extract was about 1 g/L; however, EIS results showed a similar trend for inhibition efficiency by an insignificant change. Based on the electrochemical tests, the adsorption isotherm was the Langmuir model. The *OO*L extract molecules acted as a mixed inhibitor. The Tafel polarization data at the temperature range of 298–313 K showed that the increasing temperature was not an effective factor to decrease the inhibition efficiency of the utilized inhibitor; however, the gravimetric investigation displayed that the time of 100 h resulted in a reduction of inhibition behavior. FESEM images showed that the deterioration of the corroded steel surface after 72 h in the acidic solution with 1 g/L OO*L* extract reduced significantly. In addition, the roughness of the corroded surface after 1 h decrease from 38.5 to 19.8 nm, based on the AFM images, since the OO*L* extract acted as a physical barrier for corrosive ion diffusion. EDS, FTIR-ATR, and GIXRD patterns demonstrated that the corroded surface of steel with and without inhibitor contained a mixture of FeO(OH), Fe_2_O_3_, and FeCl_2_.4H_2_O; however, the thickness of this layer and the water uptake at the surface reduced significantly. Based on experimental results, a proposed inhibition mechanism consisted of the physical adsorption of inhibitor molecules from the N or O side at the steel surface and create a barrier layer for H^+^ ions as the disrupting factor for the cathodic reaction. Besides, Cl^−^ ions adsorbed at the steel surface, and the protonated molecules of inhibitor were absorbed into these anions. In this situation, the utilized inhibitor acted as the disrupting factor for the anodic reaction.

## Declarations

### Author contribution statement

Haneih Mobtaker: Conceived and designed the experiments; Performed the experiments; Analyzed and interpreted the data.

Mahboobeh Azadi: Conceived and designed the experiments; Analyzed and interpreted the data; Wrote the paper.

Maryam Rassouli: Performed the experiments; Analyzed and interpreted the data; Contributed reagents, materials, analysis tools, or data.

### Funding statement

This research did not receive any specific grant from funding agencies in the public, commercial, or not-for-profit sectors.

### Data availability statement

The data that has been used is confidential.

### Declaration of interests statement

The authors declare no conflict of interest.

### Additional information

No additional information is available for this paper.

## Appendix A Supplementary data

supplimentary supplimentary
