# Supplementary figures and images for: The corrosion inhibition of carbon steel in 1 M HCl solution by Oestrus ovis larvae extract as a new bio–inhibitor
Source: Heliyon. 2022 Dec 17;8(12):e12297. doi: 10.1016/j.heliyon.2022.e12297 (PMC9793283; doi:10.1016/j.heliyon.2022.e12297)

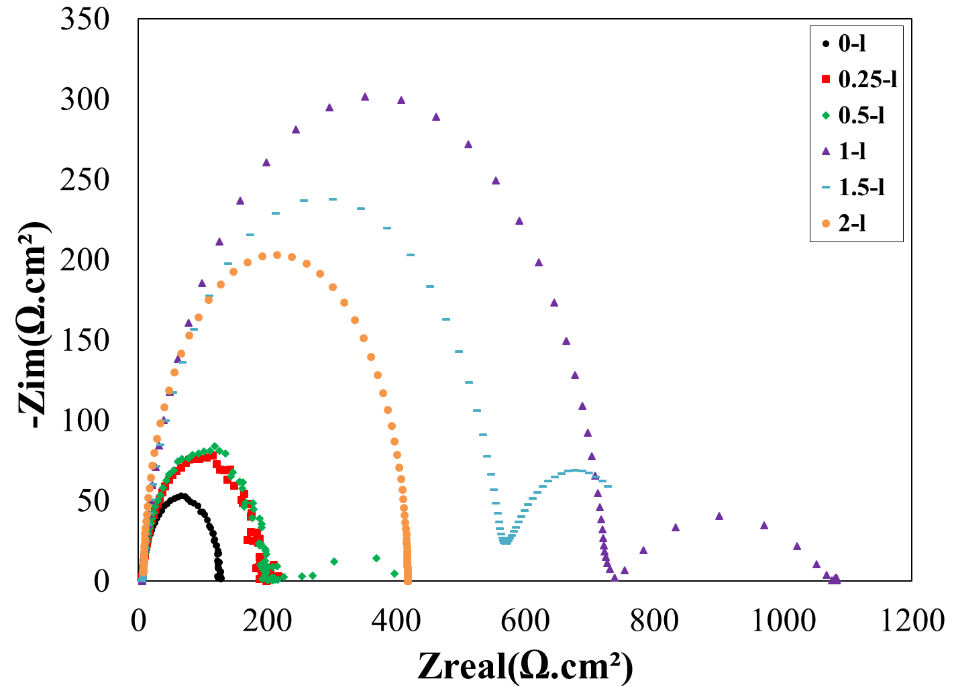


Figure S1- Nyquist plots for all specimens

Supplement: supplimentary [file mmc1.docx]
